# Body ownership and the absence of touch: approaching the rubber hand inside and outside peri-hand space

**DOI:** 10.1007/s00221-018-5361-9

**Published:** 2018-09-15

**Authors:** M. Smit, J. T. H. Brummelman, A. Keizer, M. J. van der Smagt, H. C. Dijkerman, I. J. M. van der Ham

**Affiliations:** 1 Department of Experimental Psychology, Helmholtz Institute, Utrecht University, Utrecht, The Netherlands; 2 Department of Psychiatry, University Medical Center Utrecht, Utrecht, The Netherlands; 3 Department of Health, Medical, and Neuropsychology, Leiden University, Leiden, The Netherlands

**Keywords:** Rubber hand illusion, Body ownership, Expectation, Touch, Visuo-tactile prediction, Peripersonal space

## Abstract

It is widely accepted that the integration of visual and tactile information is a necessity to induce ownership over a rubber hand. This idea has recently been challenged by Ferri et al. (Proc R Soc B 280:1–7, 2013), as they found that sense of ownership was evident by mere expectation of touch. In our study, we aimed to further investigate this finding, by studying whether the mere potential for touch yields a sense of ownership similar in magnitude to that resulting from actually being touched. We conducted two experiments. In the first experiment, our set-up was the classical horizontal set-up (similar to Botvinick and Cohen, Nature 391:756, 1998). Sixty-three individuals were included and performed the classical conditions (synchronous, asynchronous), an approached but not touched (potential for touch), and a ‘visual only’ condition. In the second experiment, we controlled for differences between the current set-up and the vertical set-up used by Ferri et al. (Proc R Soc B 280:1–7, 2013). Fifteen individuals were included and performed a synchronous and various approaching conditions [i.e., vertical approach, horizontal approach, and a control approach (no hands)]. In our first experiment, we found that *approaching* the rubber hand neither induced a larger proprioceptive drift nor a stronger subjective sense of ownership than asynchronous stimulation did. Generally, our participants gained most sense of ownership in the synchronous condition, followed by the visual only condition. When using a vertical set-up (second experiment), we confirmed previous suggestions that tactile expectation was able to induce embodiment over a foreign hand, similar in magnitude to actual touch, but *only* when the real and rubber hand were aligned on the vertical axis, thus along the trajectory of the approaching stimulus. These results indicate that our brain uses bottom-up sensory information, as well as top-down predictions for building a representation of our body.

## Introduction

Body ownership is the feeling that your body belongs to you. This feeling of ownership is achieved through integration of visual, tactile and proprioceptive information (Botvinick 2004; Botvinick and Cohen 1998; Ehrsson 2012; Tsakiris 2017) and can be experimentally manipulated using the ‘rubber hand illusion’ (RHI) (Botvinick and Cohen 1998 for a detailed procedure). It is widely accepted that the integration of visual and tactile information is a necessity to induce the rubber hand illusion (Botvinick and Cohen 1998). However, Ferri et al. (2013) conducted an experiment that challenges this idea. In their study, the mere expectation of being touched was used to try to induce a sense of ownership over the rubber hand. Their rationale was that sense of ownership is not only a bottom-up process of sensory input, but also depends on top-down influences. Based on previous experiences, our brain generates predictions about forthcoming events or stimuli (Engel et al. 2001). Ferri et al. (2013) hypothesized that the expectation of someone touching the rubber hand is enough to induce ownership over a fake rubber hand. In their set-up, the rubber hand was placed above the real hand. The rubber hand and the real hand were not touched in this experiment, but approached slowly from above by the experimenter’s hand. Results on the explicit measure (i.e., questionnaire) showed that indeed the mere expectation of being touched enabled a subjective sense of ownership over the rubber hand. Additionally, physiological measures [i.e., skin-conductance responses (SCR)] revealed that this effect was most apparent when the approaching stimulus entered the so-called ‘peripersonal space’ (Ferri et al. 2013) as opposed to extrapersonal space. Thus, Ferri et al. (2013) showed that when a stimulus (i.e., experimenter’s hand) enters the peripersonal space (i.e., near hand space), even *expectation* of touch led to ownership over a rubber hand.

This makes sense, since sensory stimuli in peripersonal space are perceived differently than those in extrapersonal space (far space). Research has demonstrated a dynamic and close relation between visual and tactile stimuli in the peripersonal space (Graziano et al. 1997; Rizzolatti et al. 1981); that is, the multisensory areas in the brain appear to code them in the same way. This was first described in monkeys, where bimodal neurons in the premotor and parietal areas (multisensory areas) respond both to tactile stimuli on the monkey’s limb and visual stimuli nearby the limb (Graziano et al. 1997; Rizzolatti et al. 1981). Human behavioral studies yield similar results: Facilitatory effects of tactile processing have been documented when visual attention was directed towards a location close to the tactile target (i.e., vibration on skin) (Driver and Spence 1998; Macaluso and Maravita 2010), again this was in near space. Even Kandula et al. (2015) showed that when an arm pointed towards the cheek and was followed by a tactile vibration on that cheek, individuals were faster than when the hand pointed away from the cheek. Cléry et al. (2015a) found similar results of enhanced tactile sensitivity with looming stimuli passing the face. These results suggest a visuo-tactile predictive mechanism, where expectation of touch in near space yields faster responses (see review Cléry et al. 2015b), and that this integration of spatial and temporal signals may be involved in the same neural networks as multisensory integration is involved (Cléry et al. 2017). Moreover, Dong et al. (1996) reported monkey parietal neurons to respond both when their face was being touched and when a ‘harmful’ stimulus was held in their peripersonal space (without touching). Therefore, these multisensory brain areas will respond to a visual stimulus that enters one’s peripersonal space (without touch) in the same way as when one is actually being touched. That is, these areas also respond to the mere expectation of touch. We know that integration of tactile and visual information has been deemed to be responsible for ownership over a rubber hand. As a consequence, if multisensory areas do respond similar to mere expectation of touch, then expectation of touch should also induce ownership over a rubber hand. Ferri et al. (2013) confirm this hypothesis. The authors interpreted this finding by suggesting that the sense of ownership was induced by the process of actively produced top-down predictions about forthcoming stimuli, which was based on the idea that sense of ownership is not only a bottom-up process of sensory input, but also depends on top-down influences.

However, although the finding of Ferri et al. (2013) is intriguing in itself, the set up precluded a direct comparison with bottom-up sensory input as it did not make direct comparisons with the conditions that reflect bottom-up processes (e.g., synchronous and asynchronous condition), which are typically used in classical RHI set-ups (e.g., Botvinick and Cohen 1998). There are some other important differences between the set-up used by Ferri et al. 2013 and classical RHI set-ups. In the study of Ferri et al. (2013), the real hand of the participants was placed *underneath* the rubber hand, instead of next to the rubber hand. Thus, when the experimenter approached the rubber hand, the real hand was also being approached. In the current study, using the horizontal set-up, where only the rubber hand is being approached, we aim to test whether approaching the hands simultaneously is a critical factor in embodying a foreign arm. Furthermore, the experimenter’s hand approached the rubber hand slowly *from above* but in lateral view of the participant (i.e., right side), instead of from up front. Related to this, participants in Ferri et al.’s (2013) study had a *shorter visual exposure* to the rubber hand than in classical RHI studies, as participants were instructed to visually follow the experimenter’s hand moving downwards. Inspired by the effect found by Ferri et al. (2013), we adapted their experiment to match the classical rubber hand set-up (see Botvinick and Cohen 1998 for a detailed procedure; set-up adopted from Kammers et al. 2009), with the only difference being actually touched versus expecting to be touched. Thus, in the current study, the rubber hand was placed *next* to the participant’s actual hand and we approached the rubber hand from the front, instead of from above. This allowed continuous visual exposure to the rubber hand as well as assessing the sense of ownership over the rubber hand using classical ownership outcome measures (i.e., proprioceptive drift and subjective embodiment). Furthermore, it also enabled us to compare classic multisensory RHI conditions (i.e., synchronous and asynchronous touch condition) to the mere expectation of touch (i.e., hereafter referred to as the predictive condition). We hypothesized that participants would experience ownership over the rubber hand in the synchronous condition as well as in the predictive condition, but not in the asynchronous (control) condition. There is general consensus about the asynchronous condition being a control condition (i.e., no ownership over the rubber hand) (Botvinick and Cohen 1998; Tsakiris and Haggard 2005), but see Rohde et al. (2011) for a different appraisal of this idea. We also included a visual only condition in which participants merely viewed the rubber hand lying in front of them, without the expectation of being touched or actual touch. Since top down predictions about forthcoming stimuli depend on previous experience (Engel et al. 2001), we further explored whether a previous experience with the RHI modulated sense of ownership in the predictive condition. Half of the participants started with the synchronous stroking condition and the other half with the predictive condition.

## Methods: experiment 1

### Participants

We tested 65 (28 females) neurologically healthy participants. Average age was 43.7 years [standard deviation (SD) = 11.6]. We recently showed that handedness does not modulate sense of ownership in the RHI (Smit et al. 2017), therefore both left and right-handed individuals were included in the current study. Participants were unaware of the purpose of the experiment. They were tested individually and in a laboratory setting inside a museum. It has to be noted that more experimental set-ups were in the same test-room. To minimize distraction, we used large occluders between the set-ups and to keep noise at minimum, we instructed participants to be as quiet as possible. This study was part of Science Live, an innovative research programme of Science Centre NEMO in Amsterdam, where participants were recruited and participated on a voluntary basis for which ethical approval was obtained prior to the study. The study was conducted in accordance with the standards of the declaration of Helsinki and was approved by the local ethical committee. Written informed consent was obtained prior to participation.

### Design

**Fig. 1:**
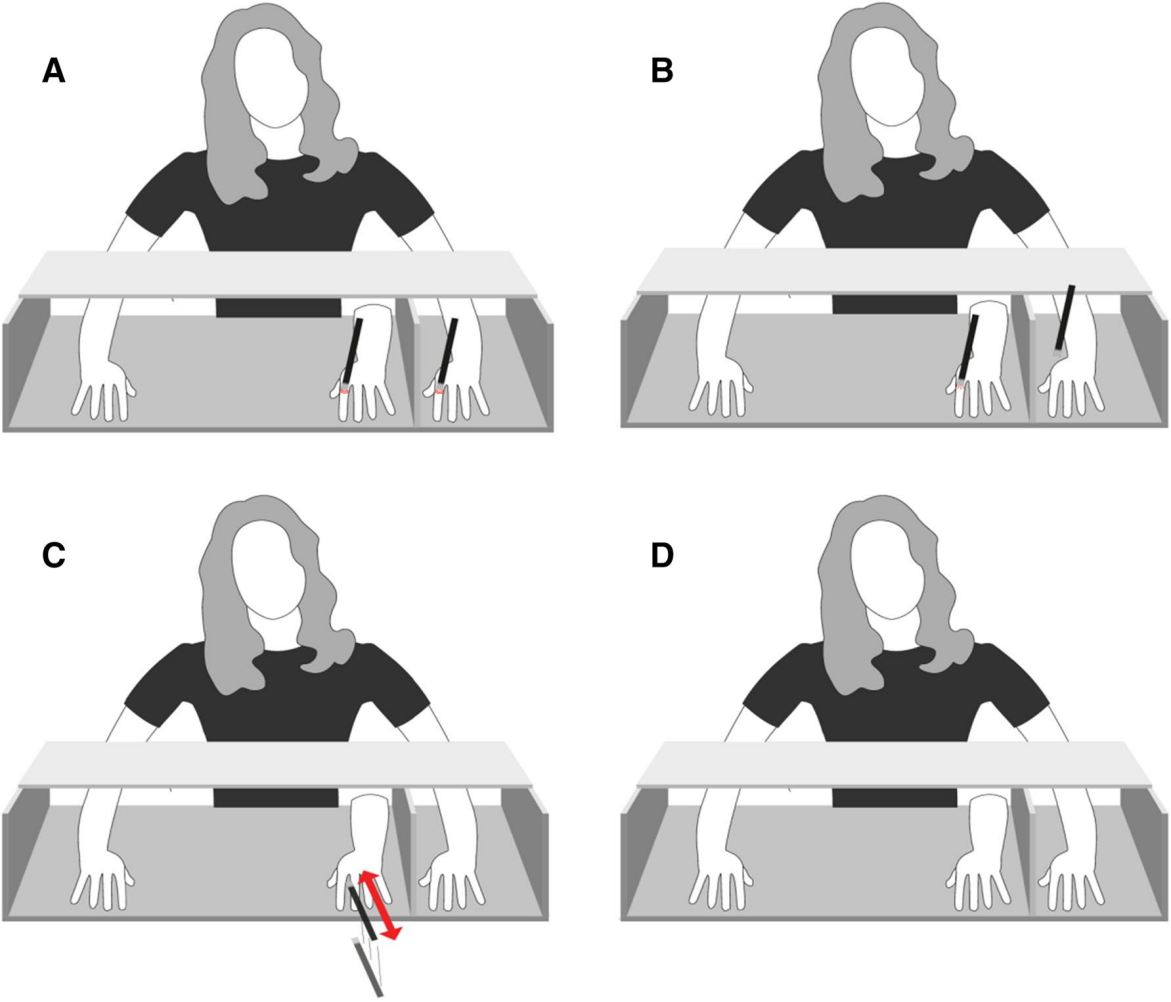
**a** Synchronous condition, **b** asynchronous condition, **c** predictive condition, **d** visual-only condition. Note that participants did not see their real left hand and that positioning of the hands of the participants was similar in all conditions. To optimize the illusion, a black cloth (not shown in figure) was placed over the shoulder of the participant, which prevented visual feedback of attachment of the own and/or rubber hand to the body

The experiment consisted of four conditions in a within subjects design (i.e., synchronous, predictive, asynchronous, visual only, see Fig. 1 and “Procedure and measurements” and “Stimulation” for details). The experiment was performed in a block-randomized design, in which the experimental conditions were performed first and were then followed by the two control conditions. Half of the participants started with the synchronous condition and the other half with the potential of touch (i.e., predictive) condition. The order of both control conditions alternated as well (see Table 1).

**Table 1:** Overview of the experimental design

Sequence	Experimental conditions		Control conditions	
	Condition 1	Condition 2	Condition 3	Condition 4
1	Synchronous	Predictive	Visual only	Asynchronous
2	Synchronous	Predictive	Asynchronous	Visual only
3	Predictive	Synchronous	Visual only	Asynchronous
4	Predictive	Synchronous	Asynchronous	Visual only

The conditions were block-randomized, with each participant starting with one of the experimental conditions and ending with both control conditions

#### Experimental set-up

The experimental set-up consisted of a wooden box divided in two compartments. The hands of the participants were placed near the sides, with the stimulated left hand being occluded from view (see Fig. 1). The rubber hand was placed visibly in the middle part of the box. The distance between the stimulated hand and the rubber hand was approximately 17 cm. The right unstimulated hand also remained visible during the illusion. In addition to a screen that divided the stimulated hand from the rubber hand and the unstimulated hand, a black cloth was placed over the arms of the participants to make the end of the rubber hand and the participant’s left arm invisible.

### Stimulation

#### Rubber hand illusion

For all participants, all conditions were performed on the left hand for 60 s. Previous research in our lab deemed 90 s of stimulation to be appropriate to successfully differentiate between the synchronous and asynchronous condition. For feasibility reasons, we investigated the optimal time window to successfully differentiate between the synchronous and asynchronous conditions. Hence, prior to actual testing, we investigated (*n* = 20) whether there were any differences in sense of ownership after 30, 60 and 90 s of stimulation. Results indicated that the experience of the illusion, as measured with a questionnaire and proprioceptive drift (see Appendix 1) did not significantly change as stimulation time increased. Moreover, we found that time windows of 60 and 90 s successfully differentiated between synchronous and asynchronous condition. Thus, for feasibility reasons, we applied a 60-s stimulation window in Science Live science center NEMO. See Appendix 1 for details.

#### Stroking conditions

There were four conditions (Table 1 for order, and Fig. 1 for set-up). In the synchronous condition (Fig. 1a), the rubber hand and the real hand of the participant were stroked synchronously in identical stroke frequencies varying from one stroke per second to one stroke per 3 s. In the asynchronous condition (Fig. 1b), the rubber hand and real hand were stroked sequentially in an identical pattern wherein the brush only touched one hand at a time. In the predictive condition (Fig. 1c), only the rubber hand and not the real hand, was *approached* from the front and above (red arrow in Fig. 1c), but not touched. The approach movement went back and forth (two-sided arrow) once per second and varied in velocity and location to reduce habituation effects. In the visual-only condition (Fig. 1d), participants had to look at the rubber hand.

### Procedure and measurements

Prior to the experiment informed consent and demographic information was obtained. First, the hands of the participants were placed in the box and participants were instructed to keep their hands still during the whole experiment (see Fig. 1 for positioning). The wooden lid was placed over the box (not shown in Fig. 1), which occluded the actual hands and rubber hand from top view. Thereafter the experiment started. The order of measurements was as follows: (1) proprioceptive drift pre-session, (2) administration of 60 s stimulation (i.e., synchronous, predictive, asynchronous or visual), (3) proprioceptive drift post-session after *each* of the aforementioned condition, and (4) embodiment questionnaire.

#### Proprioceptive drift

Proprioception or position sense of the left index finger was obtained twice, before and immediately after the stroking. To assess the first measurement of proprioception, participants were instructed to close their eyes preventing visual feedback of arm position. A wooden lid was placed on the box, which covered the participant’s own hands and rubber hand. The participants were instructed to open their eyes and the experimenter moved his index finger alongside the top of box. Participants had to indicate (by saying stop) when the experimenter’s finger was at the *felt position* of the (left) real index finger. It took approximately 30 s between participants closing their eyes, opening their eyes and verbally reporting their estimation. The experimenter measured the felt position, and the real position. A tape measure that was attached to the back of the set-up allowed for measuring (in centimeters) the felt position and the real position of the center point of the index finger of the participant. The experimenter then removed the top cover of the box for the next trial (either approaching or multisensory stimulation). Participants were instructed to look at the rubber hand, after which one of the four stimulation conditions (i.e., synchronous, predictive, asynchronous and visual) was applied. After stimulation the participants had to close their eyes again, until the top cover was placed on the box. Thereafter participants were instructed to open their eyes again and the post-session of proprioception was obtained. The difference between pre- and post-session is indicative of how much the stimulated hand ‘drifted’ towards the rubber hand. Subsequently, a subscale of an embodiment questionnaire was administered (see below).

#### Embodiment questionnaire

To indicate the subjective sense of ownership, participants filled out the ‘ownership subscale’ of an embodiment questionnaire (adapted from Longo et al. 2008) that contained the following five items: (1) it seemed like I was looking directly at my own hand, rather than at the rubber hand; (2) it seemed like the rubber hand began to resemble my own hand; (3) it seemed like the rubber hand belonged to me; (4) it seemed like the rubber hand was my hand; (5) it seemed like the rubber hand was part of my body. The questionnaire was administered on top of the RHI box, preventing visual exposure of the rubber hand. Participants indicated their response with a pencil on a vertical visual analog scale (VAS) ranging from ‘totally agree’ (top) to ‘totally disagree’ (bottom). A cut-off score was determined based on Longo et al.’s (2008) embodiment questionnaire, and was set on the fifth step (+ 1). If participants scored on average above the cut-off score, then it is fair to conclude that the illusion was induced successfully.

### Analyses

For proprioceptive drift, we used baseline corrected difference scores (in cm); we subtracted the felt position before the illusion from the felt position after the illusion. A positive value indicated proprioceptive drift towards the rubber hand, while a negative value indicated that the participant drifted away from the rubber hand. Scores on the Embodiment Questionnaire statements were measured in millimeters (mm) and then converted into percentages. All five statements were averaged. A higher percentage score on the questionnaire represented a higher subjective sense of ownership. Since the assumption of normality was violated for especially the questionnaire measures (discussed below), we used non-parametric tests and presented box plots (medians) for both outcome measures. We used Related Samples Friedman’s Analyses of Variance (hereafter Friedman analyses), and subsequent pairwise comparisons (6) with adjusted *p* values (Bonferroni corrected), to test differences between the synchronous, predictive, asynchronous and visual conditions. If not stated otherwise, alpha levels of 0.05 (two-tailed) were used for the statistical tests. MATLAB was used to generate box plots. SPSS and JASP were used for statistical analyses.

## Results

### Ownership questionnaire

**Fig. 2:**
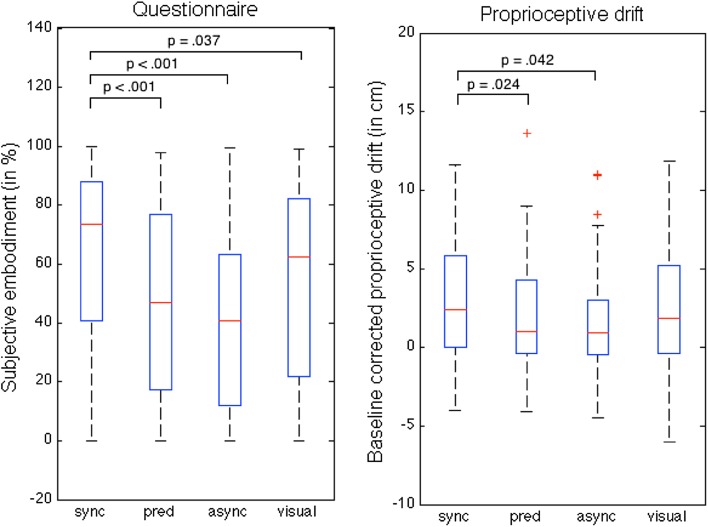
Left panel shows median subjective sense of ownership (in %) and right panel shows the median proprioceptive drift for the synchronous (sync), predictive (pred), asynchronous (async) and visual-only (visual) condition. Top lines indicate significant differences between conditions. *p* values are Dunn–Bonferroni corrected (six comparisons per outcome measure). Whiskers represent the data range; minimum and maximum within 1.5 inter quartile range (IQR). The + symbols indicate extreme outliers (> 1.5 × IQR). Note that the scales differ because different outcome measures are displayed

For the subjective sense of ownership, Shapiro–Wilk test for normality revealed that all conditions differed significantly from a normal distribution, all *p* values ≤ 0.001. Therefore, non-parametric tests were performed. Friedman’s analyses revealed an effect of condition (*χ*^2^(4) = 28.129, *p* < 0.001). Post hoc pairwise comparisons for Friedman analyses (Dunn–Bonferroni corrected) showed, as expected, a difference between the synchronous and asynchronous condition (*z* = 1.107, *p* < 0.001). In fact, post hoc testing revealed that the synchronous condition differed significantly from *all* the other conditions (asynchronous, predictive and visual), all tests *z* ≥ 0.639, *p* ≤ 0.037 for each comparison (see Fig. 2 for individual corrected *p* values, left panel). Further testing revealed no difference between the predictive and asynchronous conditions (*z* = 0.098, *p* = 1.000), the predictive and visual conditions (*z* = − 0.369, *p* = 0.688) nor the asynchronous and visual conditions (*z* = − 0.467, *p* = 0.274).

### Proprioceptive drift

For the proprioceptive drift, Shapiro–Wilk test for normality revealed that the predictive (*p* = 0.036) and asynchronous (*p* = 0.002) conditions differed significantly from a normal distribution. Therefore, for the PD measure, non-parametric tests were performed as well. A Related Samples Friedman’s Analyses of Variance again revealed an effect of condition, *χ*^2^(3) = 10.954, *p* = 0.012. Post hoc pairwise comparisons for Friedman analyses (Dunn–Bonferroni corrected) revealed a significant difference between the synchronous and asynchronous conditions (*z* = 0.631, *p* = 0.042), and the synchronous and predictive conditions (*z* = 0.672, *p* = 0.024). Surprisingly, and in contrast to the subjective experience (questionnaire), there was no statistical difference between the synchronous and visual conditions (*z* = 0.303, *p* = 1.000). In addition, further testing revealed neither a difference between the predictive and asynchronous conditions (*z* = − 0.041, *p* = 1.000), the asynchronous and visual conditions (*z* = − 0.328, *p* = 0.964) nor the predictive and visual conditions (*z* = − 0.369, *p* = 0.688).

### Correlations between the embodiment questionnaire and the proprioceptive drift

We used Kendall’s tau statistic to test whether more explicit accounts of the illusion (the embodiment questionnaire) correlated with the implicit measure of the illusion (position sense of the left index finger). We found a significant positive correlation between the embodiment questionnaire and the proprioceptive drift for the sync condition *τ*_b_ = 0.296, *p* < 0.001, the pred condition *τ*_b_ = 0.412, *p* < 0.001, the async condition *τ*_b_ = 0.305, *p* < 0.001 and the visual condition *τ*_b_ = 0.390, *p* < 0.001. This relation indicated that in all conditions, the larger the participants’ position sense drifted to right (towards the rubber hand) the higher they rated the subjective experience of the illusion.

### Previous experience and the potential for touch

We further explored whether a previous experience of touch in the RHI modulated the sense of ownership in the predictive condition. Half of the participants started with the synchronous stroking condition and the other half with the predictive condition. We found no difference between the predictive condition on position 1 and position 2 (i.e., after the synchronous condition), hence we found no effect of previous multisensory experience on both outcome measures. Likewise, for the synchronous condition, order of condition also did not matter, see Table 2 for statistics.

**Table 2:** Statistics of both outcome measures [i.e., questionnaire (Q) and proprioceptive drift (PD) for the experimental conditions predictive (pred) and synchronous (sync) on position 1 (P1) or position 2 (P2)]

Condition 1	Condition 2	*W*	*p*	95% confidence interval	
				Lower	Upper
predQ P1	predQ P2	246.0	0.792	− 20.385	25.855
predPD P1	predPD P2	208.5	0.629	− 2.600	1.950
syncQ P1	syncQ P2	280.0	0.542	− 10.715	16.865
syncPD P1	syncPD P2	221.0	0.604	− 2.900	1.650

*W* Wilcoxon signed-rank test

### Control for sequence effects

We block randomized the conditions (Table 1); the two control conditions (i.e., visual only, asynchronous) were always positioned at the end. As a result, this could cumulatively impact both the outcome measures, that is, the more exposure time the more the illusion is experienced in the visual and the asynchronous conditions. Therefore, additional data (*n* = 28) were gathered where the control conditions were positioned as the first two conditions (*n* = 14 asynchronous on first position visual only on second position, and n = 14 vice versa) and compared to the last two positions (positions 3 and 4) of the original sample discussed above (*n* = 33, *n* = 30, respectively). Individual Mann–Whitney *U* tests (for between pairs, e.g., position 1 versus 3) and Wilcoxon rank tests (for within pairs, e.g., position 1 versus 2) were performed to compare the relevant pairs of conditions. No effect of order was found on proprioceptive drift or ownership questionnaire scores for neither the between nor within tested pairs, *all Z* statistics ≤ − 0.1651, all *p* values ≥ 0.099.

## Discussion: experiment 1

Results of experiment 1 revealed that *approaching* the rubber hand without touching it did not induce a larger proprioceptive drift or subjective sense of ownership than asynchronous stroking of the rubber hand. In addition, a previous experience with the RHI did not modulate sense of ownership in the predictive condition. Generally, our participants gained most sense of ownership in the synchronous condition, followed by the visual-only condition. These two conditions did not differ significantly in the (implicit) drift measure. In the set-up of Ferri et al. (2013), the experimenter’s hand was entering the peripersonal space of the real hand since this hand was placed *underneath* the rubber hand and thus in line with the trajectory of the approaching hand. It could be that in our set-up the anatomical (spatial) mismatch between the real and the rubber hand (positioned far apart, with the experimenter’s hand only approaching the rubber hand) disrupted the sense of ownership instead of facilitating it. Critically, Ferri and Costantini (2016) wrote a specific commentary on this matter. Here, the authors stated that to induce ownership by mere expectation, the approaching movements should be directed towards both hands. It seems that it is this methodological difference (vertical instead of horizontal) that is critical and drives the effect of mere expectation. To directly test this, we performed the experiment again with four different conditions. For the conditions of main interest, we used our original set-up in the horizontal plane, i.e., rubber hand lateral to the real hand, (replication of experiment 1) *and* one on the vertical plane (i.e., rubber hand above the real hand). Thus, the latter, vertical set-up was analogous to the set-up of Ferri et al. (2013), except for the fact that we used 60 approaching movements (procedure identical to our experiment 1). To investigate the difference between tactile expectancy and actual touch, we also used the classical rubber hand set-up (synchronous actual stroking with rubber hand lateral to the real hand). Including this condition allowed us to check whether the participants were susceptible to the illusion (positive control), as it is such a robust and replicable effect (Tsakiris 2017; Kilteni et al. 2015). We also added a vertical condition where only the rubber hand was approached. The real hand of the participant was on the back, thus testing whether both rubber and own hand need to be in the approach trajectory. Overall, we expected that actual touch induced most ownership over the foreign hand. Because of the spatial alignment of both the real and the rubber hand, we also expect embodiment over a rubber hand in the new vertical set-up, and no embodiment in both the horizontal set-up (replication of experiment 1) and the vertical set-up where the real hand was not present. Like Ferri et al. (2013), we administered the questionnaire of Longo et al. (2008), which consisted of the components ‘embodiment’, ‘loss of own hand’, ‘movement’ and ‘affect’.

## Experiment 2

### Methods

#### Participants

We tested 16 (11 females) neurologically healthy participants. One participant was formally diagnosed with idiopathic sleeping hypersomnia and reported this during testing because of experienced drowsiness and was excluded from the study. Average age of the final inclusion (15) was 22.60 years (SD = 3.22). All individuals were right-handed by self-report. Participants were tested individually and were unaware of the purpose of the experiment. The study was conducted in accordance with the standards of the declaration of Helsinki and was approved by the local ethical committee. Written informed consent was obtained prior to participation.

#### Design: experiment 2

The experiment consisted of four conditions in two different set-ups within subjects design, i.e., synchronous horizontal (syncH), predictive horizontal (predH), predictive vertical (predV) and a predictive vertical control (predVC) condition with no hand in close proximity of the approached rubber hand (i.e., hands were on participants back). Unlike the first experiment, each condition was administered twice in a block-randomized design.

#### Experimental set-up: experiment 2

For the syncH and predH, the experimental set-up was identical to experiment 1 except for placement of the right real (unstimulated) hand; to keep the vertical and horizontal set-up similar, the right real (unstimulated) hand rested on each participant’s lap. The predV set-up consisted of a black box (9 cm in height), with a left rubber hand on top of the box and the participant’s real left hand exactly underneath it. The set-up of the predVC was similar to the PredV, only now the real hands were on the participants back, and thus *no* real hands were in close proximity of the approached left-handed rubber hand. Again, as in experiment 1, to optimize the illusion, a black cloth was placed over the shoulder of the participant, which prevented visual feedback of attachment of the own and/or rubber hand to the body.

#### Stimulation: experiment 2

##### Rubber hand illusion

For all participants, all conditions were performed on the left hand for 60 s (see Appendix 1 for data on this).

##### Stroking conditions

Stroking procedures of conditions syncH and predH were similar to, respectively, the synchronous and predictive conditions of experiment 1. In the predV condition, we applied the same approaching movements as in experiment 1, only now the real (unseen) left hand was *underneath* the seen rubber hand (analogous to Ferri et al. 2013). The same approaching procedure was applied in the predVC condition, only now the hands were on the back of the participant. In this case, the approaching movement was only directed to a rubber hand. In short, the approach movements in all ‘pred’-conditions were identical to the procedure in experiment 1.

##### Embodiment questionnaire

We administered the questionnaire of Longo et al. (2008), which was the same as Ferri et al. (2013). The questionnaire consisted of ten items for the component ‘embodiment’, five items for the component ‘loss of own hand’, three items for the component ‘movement’ and three items for the component ‘affect’. Participants had to rate each item on a 7-point Likert scale going from *strongly disagree* (− 3) to *strongly agree* (3), in which 0 indicates the neutral rating of “neither disagree or agree”. Again, the cut-off for experiencing subjective sense of ownership was set on the fifth step (+ 1). If participants scored on average above the cut-off score then it is fair to conclude that the illusion was induced successfully. At the end of the experiment, we had an additional question where we asked the participants to rate the overall strength of the illusion (e.g., strength in terms of ownership) for each condition, also on a scale from *very weak* (− 3) to *very strong* (3).

#### Analyses: experiment 2

Scores on the Embodiment Questionnaire items were averaged across components. First, we tested whether the observed scores were different from neutral (0). Second, we tested for differences *between* the four conditions. We applied the same procedure for both questionnaires. For questionnaire 1, data approximated normality and therefore parametric tests were used (discussed below). For the second question, data violated a normal distribution and therefore we present a table with medians. In the case of parametric data, we used a repeated-measures ANOVA (using Jasp software) and in case of non-parametric data we used Friedman analyses (using Jamovi software). Subsequent pairwise comparisons were Bonferroni corrected. If not stated otherwise, alpha levels of 0.05 (two-tailed) were used for the statistical tests.

## Results: experiment 2

### Embodiment questionnaire

**Fig. 3:**
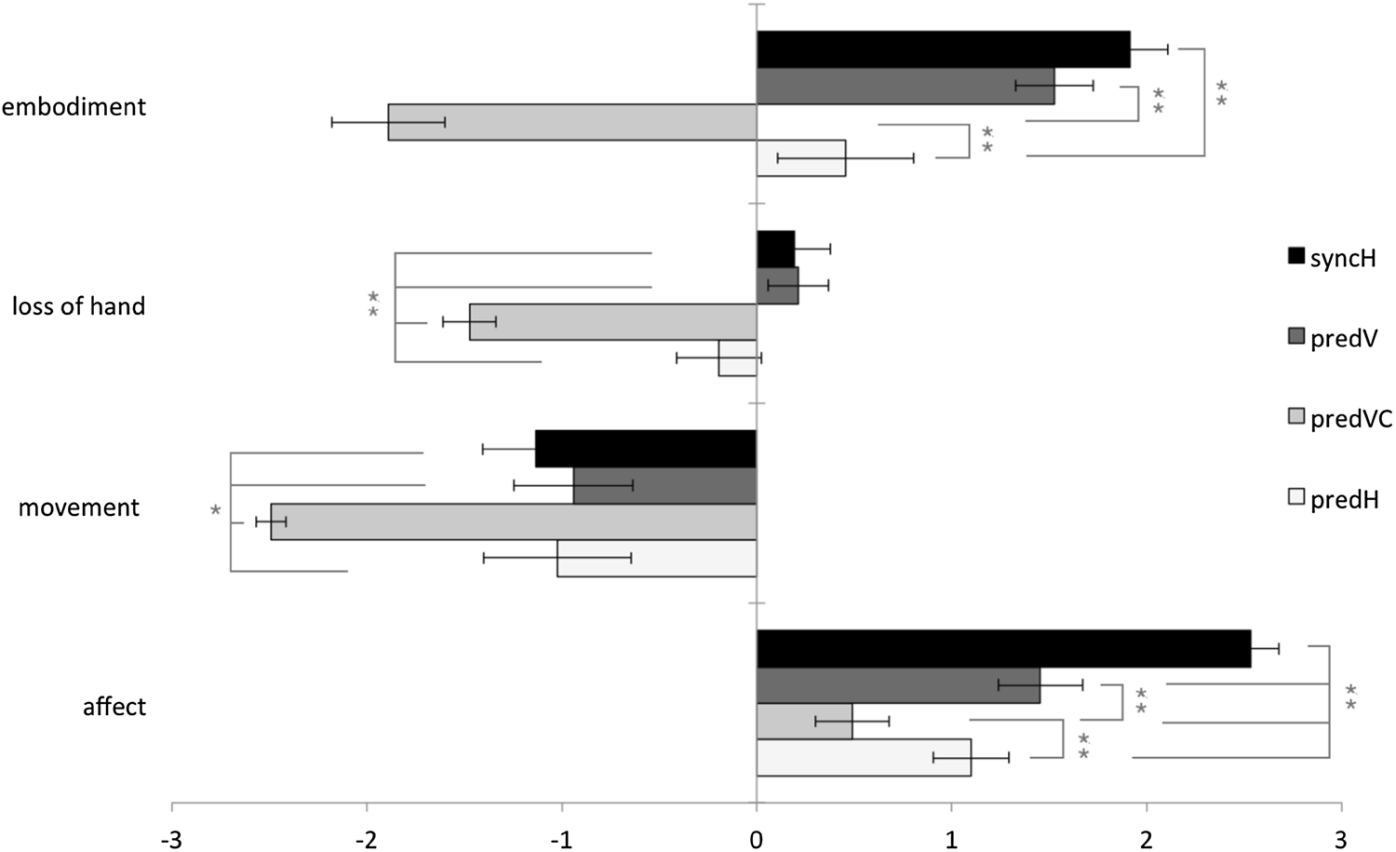
Average subjective ratings of the embodiment questionnaire on the components embodiment, loss of hand, movement and affect for the synchronous horizontal (syncH), predictive vertical (predV), predictive vertical control (predVC) and predictive horizontal (predH) condition. Lateral lines indicate significant differences between conditions (*p ≤ 0.028; **p ≤ 0.002). *p* values are Bonferroni corrected. Error bars represent standard error of the mean

For the subjective sense of ownership, Shapiro–Wilk test for normality revealed data approximated normality for all components; only the predVC of the embodiment component (*p* = 0.004), and the syncH of the affect component (*p* = 0.004) were not normally distributed. All the other 14 ‘conditions’ (4 conditions per component) were normally distributed (range *p* value = 0.067 to *p* = 0.916), therefore parametric tests were used. To facilitate comparison with results of Ferri et al. (2013), we present means for all the data in Fig. 3.

### Component embodiment

All, but predH differed (*t*(14) = 1.310, *p* = 0.211) significantly from zero for the embodiment component, all tests summarized *t*(14) ≥ 7.604, *p* ≤ 0.001. For PredVC, this score was negative (Fig. 3), indicating that on average participants did not embody the rubber hand when no hands were present. SyncH and PredV were positive; in these conditions, the rubber hand was embodied. Critically, the predH did not differ from zero, indicating, on average, a neutral response for the embodiment of the rubber hand. Repeated measures ANOVA was used to test for differences between conditions, and revealed an effect of condition *F*(3,42) = 53.47, *p* < 0.001. For post hoc comparisons, directly testing the two conditions of interest PredV versus PredH reveals us a marginally significant effect *t*(14) = 2.583, *p* = 0.022, indicating that in terms of expectation of touch we do find more evidence for embodiment in the vertical than in the horizontal set-up. However, we added additional (positive and negative controls) conditions (syncH and predVC), and as a consequence, this effect did not survive subsequent Bonferroni corrections *t*(14) = − 2.583, *p* = 0.130 (*pbonf*). Intriguingly, the syncH did not differ from predV *t*(14) = − 1.646, *p* = 0.732 (*pbonf*), indicating both conditions (statistically) did not differ in terms of experienced illusion. SyncH did differ significantly from predH *t*(14) = − 4.588, *p* = 0.003 (*pbonf*), and predVC *t*(14) = − 10.825, *p* < 0.001 (*pbonf*). As expected, the PredVC differed from all conditions, all tests summarized *t*(14) ≥ 7.996, *p* ≤ 0.001 (*pbonf*).

### Component loss of hand

As can be seen in Fig. 3, only the predVC control differed from zero *t*(14) = − 10.968, *p* < 0.001, indicating that loss of hand was not experienced in all conditions, all tests summarized *t*(14) ≥ 1.359, *p* ≤ 0.196, but especially not in the predVC. We found an effect of condition *F*(3,42) = 28.32, *p* < 0.001, that was mainly driven by the control predVC condition, which differed significantly from all the other conditions, all tests summarized: *t*(14) ≥ 5.340, *p* ≤ 0.001 (*pbonf*). The other conditions did not differ significantly from one another, all tests summarized *t*(14 < − 2.056, *p* > 0.353 (*pbonf*).

### Component movement

On average, the conditions were rated negatively (Fig. 3), and one sample test revealed that all conditions were significantly different from neutral, all tests summarized *t*(14) ≥ − 2.690, *p* ≤ 0.018. None of the conditions generated the subjective feeling that the hand moved to the rubber hand. Mauchly’s test indicated that the movement data violated the assumption of sphericity (*p* = 0.018), therefore degrees of freedom were corrected using Greenhouse–Geisser (*ε* = 0.738). Repeated measures ANOVA revealed an effect of condition, *F*(2.213, 30.985) = 7.714, *p* = 0.002, *η*^2^ = 0.343. When testing the difference between conditions only the control condition predVC differed significantly between all the other conditions, all tests summarized: *t*(14) ≥ 3.354, *p* ≤ 0.028 (*pbonf*). Other conditions did not differ significantly from one another, all tests summarized: *t*(14) ≤ − 0.215, *p* = 1.000 (*pbonf*).

### Component affect

All conditions significantly differed significantly from zero, all tests summarized *t*(14) ≥ − 0.593, ≤0.021, indicating that the experience was on average enjoyable and interesting, even when no hand was present. Repeated measures ANOVA showed an effect of condition, *F*(3,42) = 40.53, *p* < 0.001. The appeal was especially present for the syncH condition when testing between conditions, since syncH was different from all the other conditions, all tests summarized: *t*(14) ≥ − 6.287, *p* ≤ 0.001 (*pbonf*). PredH and predV were both different from PredVC, summarized: *t*(14) ≥ − 4.698, *p* = ≤ 0.002 (*pbonf*), but not different from each other *t*(14) = − 2.555, *p* = 0.137 (*pbonf*), indicating that on average and statistically, participants did not differentiate between these latter conditions in terms of interest and appeal, but did find the experience more pleasant and interesting than in the control condition.

Finally, the cut-off was set at + 1, thus according to this criterion, participants only experienced the illusion in predV and the syncH, and not in all the other conditions.

### Overall illusion strength

At the end of the experiment, we asked the participants to rate the overall strength of the illusion (the extent the participant felt the rubber hand was theirs) for each condition, also on a scale from *very weak* (− 3) to *very strong* (3).

Data were not normally distributed for any of the conditions *p* ≤ 0.026, non-parametric tests were used.

One sample Wilcoxon signed-rank test revealed that all conditions differed significantly from neutral (zero), all *W* ≥ 106.500, *p* ≤ 0.006. As displayed in Table 3, participants experienced, on average, ownership in the syncH, predV, and predH, as these conditions were rated positive. Participants reported no ownership in the predVC as this condition was rated negative. Friedman analyses showed an effect of condition *χ*^2^(3) = 37.3, *p* < 0.001. Subsequent post hoc comparisons are displayed in Table 3. SyncH now differed from all conditions, and thus was rated most strong. Interestingly, predV differed also from PredH, indicating that the illusion was significantly stronger in the vertical set-up. All conditions differed significantly from the control condition (predVC).

**Table 3:** Statistics of the strength of illusion ‘questionnaire’ for the synchronous horizontal (syncH), predictive vertical (predV), predictive vertical control (predVC) and predictive horizontal (predH) condition

Condition 1	Median	Condition 2	Median	*p*	*pbonf*
syncH	3	predV	2	< 0.001	0.006
syncH	3	predH	1	< 0.001	0.001
syncH	3	predVC	− 2	< 0.001	< 0.001
predV	2	predH	1	< 0.001	0.006
predV	2	predVC	− 2	< 0.001	< 0.001
predH	1	predVC	− 2	< 0.001	< 0.001

*W* Wilcoxon signed-rank test

### Verbal reports and observations

Reactions during the syncH condition were unanimously positive and were most often accompanied by a positive affect (i.e., amusement, surprise). Interestingly, the predV evoked more reactions, but these were also more diverse, varying between participants from positive: “This feels more interesting, because my hand is underneath it”, “This is so fascinating” to slightly more adverse: “I wanted to withdraw my hand when you approached me”, “I wanted to close my eyes every time you almost touched my hand, it was an unsettling feeling” and another participant reported “Every time you approached me, I automatically pressed my own arm against the table surface to get sensations in my own hand again”. During the predH condition, participants seemed less intrigued, and the condition also elicited less reactions, these were similar to reactions in experiment one. Some participants “felt air and wind” on their own hand, a few participants reported the experience of “sensations like pins and needles” on their own hand. One participant reported “I am having three hands, while my head tries to make it one percept, I still perceive it as three where I could not move my own hand”. The predVC evoked almost no reaction; the rubber hand felt like an external object “this hand felt very alien to me”.

## Discussion: experiment 2

In this experiment, we aimed to explore possible factors that had contributed to the discrepancy in the findings of experiment 1 and those of Ferri et al. (2013). We compared the vertical set up of Ferri et al. (2013), wherein we integrated our own approaching procedure, with the classical horizontal rubber hand set-up (both synchronous actual stroking with rubber hand and approaching the rubber hand). Finally, as a control, we also added a condition where only the rubber hand was present. Here, the rubber hand was placed in vertical axis and was approached with a brush; only now no real hand was placed underneath it.

Our results revealed that on average the syncH and predV did not significantly differ from one another in most of the components, except for the affect component where actual touch was experienced as more pleasant. The latter finding was confirmed by verbal reports. We cautiously suggest that expectation or potential for touch and actual touch both elicit the illusion to a similar extent. We, however, have to note that the difference between the vertical approaching movements and the horizontal approaching movements did not survive Bonferroni corrections for the embodiment component, while actual touch consistently differed from the horizontal approaching movements. When isolating the effect of our vertical approach set-up and comparing it directly to Ferri et al.’s findings, we see a similar pattern of results, albeit the absolute magnitude is slightly smaller than the effect seen in Ferri et al. (2013) (see “General discussion”). We do agree with their commentary that to embody a fake rubber hand, touch is not a necessary component *if* there is spatial and temporal contiguity; the rubber hand and the real hand have to be along the same trajectory (see “General discussion”). When, however, the rubber hand and the real hand are spatially aligned, but not in close proximity and not along the same trajectory (as in the horizontal condition), the relationship between the event and the expectation of touch becomes less causal, and embodiment is less likely to occur (see Woods et al. 2014).

## General discussion

Ferri et al. (2013) have shown that a sense of ownership over a foreign body part can occur as a result of the expectation of touch. In our study, we performed two experiments to further investigate whether the mere potential for touch (top-down process) yielded a sense of ownership similar in magnitude to that resulting from the multisensory stimulation (bottom-up process). Inspired by the finding of Ferri et al. (2013), in experiment 1, we added an extra condition (i.e., potential for touch) to the classical rubber hand set-up (see Botvinick and Cohen 1998; set-up adopted from Kammers et al. 2009), so that the only difference between conditions is either actually *being* touched or *expecting* to be touched. Although set-ups are different, conceptually a replication of Ferri et al.’s (2013) results would mean that expectation of touch could be deemed sufficient to induce a sense of ownership over a foreign body part. Our results in experiment 1 revealed that *approaching* the rubber hand without touching it did not induce a larger proprioceptive drift or subjective sense of ownership than asynchronous stroking of the rubber hand. In general, our participants gained most sense of ownership in the synchronous condition, followed by the visual-only condition. These two conditions did not differ significantly in the (objective) drift measure. Interestingly, Rohde et al. (2011) reported a similar result: They also found that visual exposure made the participants’ perceived hand location drift to the rubber hand to a similar extent as the synchronous condition did. Rohde et al. (2011) stated further that “proprioceptive drift in the RHI may not be caused by synchronous stroking, but rather that its lack may be caused by asynchronous stroking in the control condition”. Their study proposes a dissociation between the proprioceptive drift measure and the questionnaire; the former is caused by visuo-proprioceptive integration, and the latter by multisensory (i.e., visual, proprioceptive and tactile) integration. Thus, for proprioceptive drift, asynchronous stroking disrupts this visuo-proprioceptive integration. Intriguingly, our results show a (positive) relation between the proprioceptive drift and the questionnaire, indicating at least partial overlap between the underlying mechanisms (see Tajima et al. 2015). However, our data also concur with Rohde et al. (2011); for proprioceptive drift to occur visuo-proprioceptive integration is deemed responsible. In our set-up, the proprioceptive alignment (i.e., the spatial alignment of the hands in anatomical similar position) plus the visual capture of the rubber hand indeed induced a drift, which was statistically not distinctive from multisensory stimulation (i.e., proprioceptive, tactile and visual information). In other words, actual touch did not add more drift than visual and proprioceptive input alone. However, proprioceptive drift in the visual condition was different compared to the asynchronous condition, hence, asynchronicity disrupted potential drift. Thus, we confirm findings of Rohde et al. (2011): For the proprioceptive drift measure, visuo-proprioceptive integration seemed to cause the drift, and asynchronicity disrupted it. In contrast, for the embodiment questionnaire, multisensory stimulation (i.e., proprioceptive, tactile and visual information) differed from all the other conditions, indicating that the effect in more explicit accounts of the illusion was actually driven by multisensory integration. Thus, as Rohde et al. (2011) suggested, for both measures different underlying mechanisms seem responsible.

One could further argue that in our set-up the ‘predictive’ condition accounting for the potential of touch, with its approaching movements, disrupted the illusion to a similar extent as the asynchronous condition did. If we take a closer look at what the predictive condition entails, we observe the same kind of phenomenon as in the asynchronous condition; in the asynchronous condition, participants expected to feel the touch that they see, but did not feel it simultaneously. The *temporal* disparity between the seen and felt touch ‘disrupted’ the illusion. In the predictive condition, participants expected to be touched, but the touch never comes, which violates the expectation of touch. Anecdotal reports during experiment 1 confirmed this; touch was expected, but never occurred, which could have disrupted embodiment. However, expectation of touch did occur in the set-up of Ferri et al. (2013). The difference in set-up seems crucial; the experimenter’s hand was entering the peripersonal (hand) space of the real hand, since this hand was placed *underneath* the foreign hand and thus in line with the trajectory of the approaching hand. In other words, in this case, it is the *spatial* disparity that seems critical; in our set-up, the spatial mismatch between the real and the rubber hand (positioned further apart, with the experimenter’s hand only approaching the rubber hand) disrupted the sense of ownership instead of facilitating it.

Ferri and Costantini (2016) wrote an insightful commentary on this specific matter. They stated that to experience embodiment over a rubber hand, tactile expectation should be generated on both the rubber hand and the real hand, in the same path or trajectory. When the own hand is outside this path, the illusion will be less vivid. The fact that tactile expectation can evoke embodiment over a foreign arm is already intriguing, but why does it differentiate between a vertical or horizontal position, more specifically why does expectation of touch only elicit a vivid illusion when the hand is within the peri-hand space and not when it is lateral to the rubber hand, but still very close? According to the Bayesian statistical inference framework, prior life experiences in sensory regularities (e.g., spatial and temporal consistency) allow us to make inferences or predictions about forthcoming events (Friston et al. 2006; Friston 2010). Our brain shapes these predictions by updating the prediction to the actual outcome (i.e., Bayesian updating). If we apply this framework to our manipulations, in our experiments, we attempted to induce a *visuo-tactile* inference, that is, a visual event, such as an approaching object towards the hand, is likely to predict (based on prior or innate experiences) a tactile consequence (i.e., it will cause a touch on the hand). Causality between the visual and tactile event is more likely to occur when temporal events (e.g., when do I feel the touch) and spatial characteristics (e.g., is that going to touch me) follow the same rules that we learned in prior experiences. Thus, touch is more likely to occur or to be predicted when the rubber and real hands are spatially aligned with the trajectory of the approaching stimulus, than when they are not positioned along the same trajectory. We tested this in our second experiment and confirmed that tactile expectation was able to induce embodiment over a foreign hand, to a similar extent as actual touch did, but *only* when the real hand was aligned with the path of the approaching stimulus. When the hand was slightly further away, i.e., lateral to the real hand, responses were not different from neutral. We also observed that approaching only a rubber hand while the real hands were anatomically misaligned to the rubber hand (i.e., on the participants back), which violated the spatial consistency, no embodiment occurred (different from neutral). We suggest that a complex interaction between the bottom-up properties of bimodal neurons (i.e., cells that respond both to visual and tactile information near the body) and higher order visuo-tactile inferences are involved in building a representation of our body.

In a recent study, Ferri et al. (2017) replicated their own findings for the questionnaire, which was their sole outcome measure for the vividness of the illusion as well. They also recorded neural activity using near-infrared spectroscopy (fNIRS). Here they found more activation in the multisensory areas, i.e., the inferior parietal cortex, contralateral to the ‘approached’ hand than when a wooden hand-like object was approached. Again, this shows that our brain does not only ‘wait’ for incoming (bottom-up) sensory stimuli to form a representation of the body, but it also generates active top-down predictions about the bodily consequences of the surrounding sensory events to the extent that these predictions can change the representation of our body. To go one step further, a recent study (Kilteni and Ehrsson 2017) found that illusions in body ownership also influenced sensory prediction. Here, the authors found that experiencing hand ownership produced somatosensory attenuation during self-touch. Thus, sensory prediction does not only influence the representation of the body (as in our case), bodily illusions also influence sensory expectations (for further reading see Kilteni and Ehrsson 2017). With respect to differences in actual touch and predictive touch, future studies should test how strong the predictive effect is compared to actual touch; does actual touch (compared to predicted touch), follow the same pattern of activation in the multisensory areas. As a side note, we found in our data that the affect component, which comprises of enjoyment, appeal and pleasantness, actual touch did differ from all the other conditions. Thus, although the rubber hand could be embodied similarly between mere expectation and actual touch, the affective component was less vivid for mere expectation. In fact, some participants found the approach movement quite unsettling, and felt like retracting their own real hand.

Thus, overall, we directly tested Ferri and Costantini’s (2016) suggestion that the space or trajectory wherein these approach movements occur is critical for the illusion to be experienced. In two experiments, we compared a set-up in which a vertically aligned rubber hand and one’s real hand were approached in the same approach movement, with a more classically set-up in which the rubber and real hands were positioned in lateral fashion and where the rubber hand was approached only. We confirmed that only when both hands are along the same approaching trajectory, the mere expectation of touch was able to induce ownership over the rubber hand. Overall, these findings confirm the original observations by Ferri et al. (2013) and the suggestion made by Ferri and Costantini (2016). When isolating the embodiment component of our vertical set-up and compare it directly to the observed effects their study of 2013, we still see a similar pattern of results, albeit the absolute magnitude of this component is slightly smaller than the effect seen in Ferri et al. (2013). However, in their most recent study, the effects on the embodiment component were also more reduced (Ferri et al. 2017). Apart from the aforementioned set-up (horizontal versus vertical) differences, we have to note other methodological differences that could account for differences. First, in our task, we approached the rubber hand approximately 60 times, whereas Ferri et al. (2013) approached the hand only four times. We concluded from earlier pilot sessions that if we varied the velocity and the potential location of touch then habituation was less likely to occur. However, the likelihood of becoming habituated is still higher when the hand is approached 60 times instead of 4 times. Second, although, we block randomized our design; the addition of an actual touch condition might have attenuated the effect in the predictive condition. Although one might argue that actual touch might facilitate mere expectation, we did not find this in our first experiment. Moreover, Ferri et al. (2013) found an effect for near space using SCR. In their set up, participants had to look at the (experimenter’s) hand approaching (from above) and not at the rubber hand in front of them, the latter being constantly looked at in the classical set-up (Botvinick and Cohen 1998). In this regard, when the approaching hand is far up, participants do not see the rubber hand in front of them. It is therefore intuitive that no ownership is present for the simple reason that the rubber hand is not in sight. When the experimenter hand (from above) moves closer to the rubber hand, the participant gradually sees something laying in front of him that resembles a real hand, but this is still in the visual periphery. Incorporating something that roughly looks like a hand to our own body scheme is more likely in this sense (Tsakiris 2017). The question remains whether peripheral vision introduced an error and the participant mistook the fake embodied hand for his or her own. It would be interesting to test whether this effect for near space could be replicated if the hands were approached from up front; in this case, visual input is kept constant. Finally, while SCR was used in their study, we used proprioceptive drift as an objective outcome measure, the latter being a somewhat direct measure of body ownership. SCR is also an interesting bodily measure, since it reflects bodily arousal, but has also been associated with emotional and affective states (Rohde et al. 2011), and might thus also reflect other processes, rather than body ownership per se. To have a behavioral, implicit measure (as opposed to the questionnaire) of the vividness of the approach movements, it would be interesting to test whether the position sense of the real hand drifted in the vertical axis, i.e., towards the rubber hand, after mere expectation of touch.

All in all, in our first experiment, we found that *approaching* the rubber hand neither induced a larger proprioceptive drift nor subjective sense of ownership than asynchronous stimulation did. Generally, our participants gained most sense of ownership in the classic synchronous condition, followed by the visual-only condition. When directly comparing the horizontal set-up of experiment 1 with the vertical set-up of Ferri et al. (2013) in experiment 2, we found that tactile expectation was able to induce embodiment over a foreign hand, similar in magnitude as actual touch, but *only* when the own hand was placed along the path of the approaching stimulus. This is in accordance with previous results (Ferri et al. 2013, 2017) and suggestions (Ferri and Costantini 2016). These results suggest that our brain uses bottom-up multisensory information, as well as top-down predictions about anticipated sensory input to represent our body or induce changes in the representation of our body.

## Conclusion

From this pilot study, we can safely conclude that the experience of the illusion, as measured both subjectively and objectively did not significantly change as stimulation time increased. Moreover, we found that time windows of 60 and 90 s successfully differentiated between synchronous and asynchronous conditions. For the 30-s window, this was the case for the questionnaire only, proprioceptive drift did not survive subsequent Bonferroni corrections. In Science Live science center NEMO, we, therefore, used 60 s of stimulation time.

## Figures and Tables

**Fig. 4:**
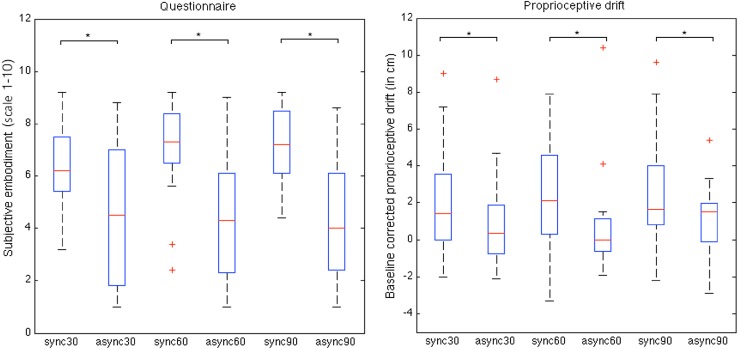
Left panel shows median subjective sense of ownership (ownership subscale only) and right panel shows the median proprioceptive drift for the synchronous (sync), and asynchronous (async) condition for all time intervals (30, 60 and 90 s). Top lines indicate significant differences between conditions. Whiskers represent the data range; minimum and maximum within 1.5 inter-quartile range (IQR). The + symbols indicate extreme outliers (> 1.5 × IQR). Note that the scales differ because different outcome measures are displayed

## Appendices

### Appendix 1

#### Aim and design pilot experiment

This study was part of Science Live, an innovative research programme of Science Centre NEMO, where participants were recruited. As such we had to limit the time per participant. Therefore, we ran a pilot study prior to the experiment in Science Live in our own lab. Here we were mainly interested in whether stimulation time could differentially impact proprioceptive drift and an embodiment questionnaire, our primary outcome measures. If so, we would choose the stimulation time that was both feasible in the Science Live test setting and that also differentiated between the classic synchronous and asynchronous conditions. Therefore, we used the classical condition (i.e., synchronous versus asynchronous) and three stimulation times (i.e., 30 s, 60 s, 90 s) and measured both proprioceptive drift and the five statements mentioned in the manuscript. Conditions were randomized for each participant.

#### Demographics

Twenty students participated in this pilot experiment. There were 5 males and 15 females, with a varying age from 20 to 25 years (*M* = 21.85; SD = 1.42). Informed consent was obtained prior to the experiment.

#### Analyses

For proprioceptive drift, we used baseline-corrected difference scores (in cm), see result section in manuscript for exact calculations. In addition, we used the same five Embodiment statements with the only difference that we used a 10-point scale (agree versus disagree), in which a cut-off score for experiencing a subjective sense of ownership was set at 5. Normality was violated especially for the questionnaire measures, and thus we used non-parametric tests and present box plots (medians) for both outcome measures. We used Related Samples Friedman’s Analyses of Variance (hereafter Friedman), and subsequent post hoc tests between synchronous and asynchronous conditions using Wilcoxon ranks test with adjusted *p* values (Bonferroni corrected). If not stated otherwise, alpha levels of 0.05 (two-tailed) were used for the statistical tests.

##### Ownership questionnaire

For the subjective sense of ownership, Shapiro–Wilk test for normality revealed that all but one condition (synchronous stroking for 60 s; *w* = 0.921, *p* = 0.012.) approached a normal distribution, all other conditions summarized: *w* > 0.927, *p* > 0.104.

As expected, Friedman’s analysis revealed an effect of synchronicity (*χ*^2^(2) = 44.085, *p* < 0.001), but no effect of stimulation time (*χ*^2^(2) = 4.560, *p* = 0.104) on the ownership questionnaire. Friedman’s analyses does not allow interactions between synchronicity and stimulation time, therefore we subtracted the asynchronous score from the synchronous score and analyzed whether this ‘true’ illusion score changed as a function of stimulated time; Friedman analyses revealed that the illusion score did not significantly change as stimulation time increased (*χ*^2^(2) = 1.658, *p* = 0.451).

Furthermore, post hoc pairwise comparisons showed a difference between the synchronous and asynchronous condition, after 30 s stimulation time (*Z* = − 3.138, *p* = 0.002, *pbonf* = 0.006), 60 s stimulation time (*Z* = − 3.827, *p* < 0.001, *pbonf* = 0.003), and 90 s stimulation time (*Z* = − 3.885, *p* < 0.001, *pbonf* = 0.003), see Fig. 4, left panel.

##### Proprioceptive drift

For the proprioceptive outcome measure, Shapiro–Wilk test for normality revealed that synchronous stroking of 90 s and both the asynchronous stroking of 30 and 60 s were not normally distributed, all tests summarized: *w* ≤ 0.926, *p* ≤ 0.013. Other conditions approached a normal distribution, all conditions summarized: *w* ≥ 0.927, *p* ≥ 0.104. As expected, Friedman’s analyses revealed an effect of synchronicity (*χ*^2^(2) = 11.655, *p* = 0.001), but no effect of stimulation time (*χ*^2^(2) = 4.179, *p* = 0.125). Therefore, we subtracted the asynchronous score from the synchronous score and analyzed whether this ‘true’ illusion score changed as a function of stimulated time; Friedman analyses revealed that the illusion score did not significantly change as stimulation time increased (*χ*^2^(2) = 0.300, *p* = 0.910). Furthermore, post hoc pairwise comparisons showed a difference between the synchronous and asynchronous condition, for 30 s stimulation time (*Z* = − 2.054, *p* = 0.040, *pbonf* = 0.12), 60 s stimulation time (*Z* = − 2.677, *p* = 0.007, *pbonf* = 0.021), and 90 s stimulation time (*Z* = − 2.496, *p* < 0.013, *pbonf* = 0.039), see Fig. 4, right panel.

## References

[CR1] Botvinick M (2004). Probing the neural basis of body ownership. Science.

[CR2] Botvinick M, Cohen J (1998). Rubber hands ‘feel’ touch that eyes see. Nature.

[CR3] Cléry J, Guipponi O, Odouard S, Wardak C, Ben Hamed S (2015). Impact prediction by looming visual stimuli enhances tactile detection. J Neurosci.

[CR4] Cléry J, Guipponi O, Wardak C, Ben Hamed S (2015). Neuronal bases of peripersonal and extrapersonal spaces, their plasticity and their dynamics: knowns and unknowns. Neuropsychologia.

[CR5] Cléry J, Guipponi O, Odouard S, Pinède S, Wardak C, Ben Hamed S (2017). The prediction of impact of a looming stimulus onto the body is subserved by multisensory integration mechanisms. J Neurosci.

[CR6] Dong WK, Hayashi T, Roberts VJ, Fusco BM, Chudler EH (1996). Behavioral outcome of posterior parietal cortex injury in the monkey. Pain.

[CR7] Driver J, Spence C (1998). Cross-modal links in spatial attention. Philos Trans R Soc B Biol Sci.

[CR8] Ehrsson HH (2012). The concept of body ownership and its relation to multisensory integration.

[CR9] Engel AK, Fries P, Singer W (2001). Dynamic predictions: oscillations and synchrony in top-down processing. Nat Rev Neurosci.

[CR10] Ferri F, Costantini M (2016). Commentary: the magnetic touch illusion: a perceptual correlate of visuo-tactile integration in peripersonal space. Front Hum Neurosci.

[CR11] Ferri F, Chiarelli AM, Merla A, Gallese V, Constantini M (2013). The body beyond the body: expectation of a sensory event is enough to induce ownership over a fake hand. Proc R Soc B.

[CR12] Ferri F, Ambrosini E, Pinti P, Merla A, Costantini M (2017). The role of expectation in multisensory body representation: neural evidence. Eur J Neurosci.

[CR13] Friston K (2010). Is the free-energy principle neurocentric?. Nat Rev Neurosci.

[CR14] Friston K, Kilner J, Harrison L (2006). A free energy principle for the brain. J Physiol.

[CR15] Graziano MS, Hu XT, Gross CG (1997). Coding the locations of objects in the dark. Science.

[CR16] Kammers MPM, de Vignemont F, Verhagen L, Dijkerman HC (2009). The rubber hand illusion in action. Neuropsychologia.

[CR17] Kandula M, Hofman D, Dijkerman HC (2015). Visuo-tactile interactions are dependent o the predictive value of the visual stimulus. Neuropsychologia.

[CR18] Kilteni K, Ehrsson HH (2017). Body ownership determines the attenuation of self-generated tactile sensations. PNAS.

[CR19] Kilteni K, Maselli A, Kording KP, Slater M (2015). Over my fake body: body ownership illusions for studying the multisensory basis of own-body perception. Front Hum Neurosci.

[CR20] Longo MR, Schüür F, Kammers MPM, Tsakiris M, Haggard P (2008). What is embodiment? A psychometric approach. Cognition.

[CR21] Macaluso E, Maravita A (2010). The representation of space near the body through touch and vision. Neuropsychologia.

[CR22] Rizzolatti G, Scandolara C, Matelli M, Gentilucci M (1981). Afferent properties of periarcuate neurons in macaque monkeys. Behav Brain Res.

[CR23] Rohde M, Di Luca M, Ernst MO (2011). The rubber hand illusion: feeling of ownership and proprioceptive drift do not go hand in hand. PLoS ONE.

[CR24] Smit M, Kooistra DI, van der Ham IJM, Dijkerman HC (2017). Laterality and body ownership: effect of handedness on experience of the rubber hand illusion. Laterality Asymmetries Body Brain Cogn.

[CR25] Tajima D, Mizuno T, Kume Y, Yoshida T (2015). The mirror illusion: does proprioceptive drift go hand in hand with sense of agency?. Front Psychol.

[CR26] Tsakiris M (2017). The multisensory basis of the self: from body to identity to others. Q J Exp Psychol.

[CR27] Tsakiris M, Haggard P (2005). The rubber hand illusion revisted: visuotactile integration and self-attribution. J Exp Psychol Hum Percept Perform.

[CR28] Woods AJ, Hamilton RH, Kranjec A, Minhaus P, Bikson M, Yu J (2014). Space, time, and causality in the human brain. Neuroimage.

